# Modifying the glycosylation profile of SARS-CoV-2 spike-based subunit vaccines alters focusing of the humoral immune response in a mouse model

**DOI:** 10.1038/s43856-025-00830-w

**Published:** 2025-04-11

**Authors:** Tyler M. Renner, Matthew Stuible, Martin A. Rossotti, Nazanin Rohani, Yuneivy Cepero-Donates, Janelle Sauvageau, Lise Deschatelets, Renu Dudani, Blair A. Harrison, Jason Baardsnes, Izel Koyuturk, Frank St. Michael, Jennifer J. Hill, Usha D. Hemraz, Anne E. G. Lenferink, Jamshid Tanha, Barbara Fernandes, Antonio Roldao, Michael J. McCluskie, Bassel Akache, Yves Durocher

**Affiliations:** 1 National Research Council Canada, Human Health Therapeutics Research Centre, Ottawa, Ontario Canada; 2 National Research Council Canada, Human Health Therapeutics Research Centre, Montreal, Quebec Canada; 3 Department of Biochemistry and Molecular Medicine, University of Montreal, Montreal, Quebec Canada; 4 Department of Biochemistry, Microbiology and Immunology, University of Ottawa, Ottawa, Ontario Canada; 5 iBET, Instituto de Biologia Experimental e Tecnológica, Oeiras, Portugal

**Keywords:** Protein vaccines, Adaptive immunity

## Abstract

**Background:**

Protein subunit vaccines have a strong track record of efficacy and safety and have been widely applied for prevention of a variety of infectious diseases. The impacts of post-translational modifications of vaccine antigens are often overlooked, despite the fact that they can vary significantly depending on the expression hosts (e.g., bacteria, yeast, plant, insect or mammalian cells) and the culture conditions used for their manufacturing.

**Methods:**

Using SARS-CoV-2 spike trimers as model antigens, we sought to evaluate the immunological impact of modulating their state of glycosylation. Spike proteins rich in complex-type (CT), high-mannose (HM) or paucimannose (PM) N-linked glycans were produced using Chinese Hamster Ovary (CHO) cells (cultured with or without the mannosidase inhibitor kifunensine) or insect cells.

**Results:**

Here we show that when these antigens are adjuvanted with liposomes composed of sulfated lactosyl archaeol (SLA), all glycoforms are highly immunogenic and induce abundant spike-specific serum IgG and IFN-γ producing T-cells within female C57BL/6 mice. The spike antigen with CT glycans induces a significantly more potent neutralizing immune response, which directly correlates to more abundant receptor binding domain (RBD)-specific IgG when comparing to the antigen with HM glycans. This observation remains true whether the spike is resistin- or T4 foldon-trimerized, indicating that the glycosylation effect is not trimerization domain-specific. Spike with PM glycans induces remarkably low titers of neutralizing antibodies and RBD-specific IgG.

**Conclusions:**

The results highlight the significant impacts of a vaccine’s antigen glycosylation profile in directing the immune response, which should be an important consideration for designing efficient protein-based vaccines.

## Plain Language Summary

Protein subunit vaccines are routinely used in the clinic. These vaccines incorporate a carefully chosen fragment, or protein subunit, of an infectious agent. The manufacturing conditions of these protein subunits are quite variable (e.g., using bacteria, yeast, plant, insect or mammalian cells), which differ in how they glycosylate, or conjugate sugar molecules, to these proteins in a process known as glycosylation. Glycosylation is key for providing the proper shape and function to many proteins, such as the SARS-CoV-2 spike protein responsible for allowing the virus to enter into cells. The effects of glycosylation patterns on vaccine function are not entirely clear. Our data shows that for SARS-CoV-2 spike, proteins with glycosylation profiles closest to those seen in humans were most effective as vaccine components. Glycosylation state may become a key consideration in designing vaccines of the future.

## Abstract (web summary)

Renner et al. evaluate the impacts of different types of glycan moieties on the immunogenicity of SARS-CoV-2 spike in a mouse model. The presence of complex type glycans correlate with increased levels of neutralizing antibodies.

## Introduction

Vaccines based on live-attenuated or inactivated preparations of viral and bacterial pathogens have been actively developed for centuries^1^. Modern advancements have enabled more refined vaccine designs, including protein subunit vaccines (SUVs) which are based on recombinantly produced antigens that correspond to a specific protein subunit of a pathogen. Protein SUVs reduce safety concerns associated with live-attenuated and inactivated vaccines and have the added advantage of a more focused immune response. Since 2006, a number of protein SUVs have been approved by regulators and are now widely used, which include Gardasil and Cervarix (against human papillomavirus, produced in yeast or insect cells, respectively), Shingrix (against herpes zoster; produced in Chinese Hamster Ovary (CHO) cells), and Arexvy and Abrysvo (against Respiratory Syncytial Virus (RSV); produced in CHO cells).

The potentially reduced immunogenicity of SUVs compared to live-attenuated or inactivated vaccines can be mitigated by formulating SUV antigens with strong adjuvants^2^. Most vaccines in the clinic today use one of a handful of adjuvants, such as aluminum salts (e.g., Gardasil), saponins (e.g., Nuvaxovid), TLR-agonists (e.g., Heplisav-B), lipid emulsions (e.g., Fluad) or a combination of these (e.g., Shingrix and Cervarix). Our group has developed sulfated lactosyl archaeol (SLA) archaeosomes as a novel protein SUV adjuvant, and tested this preclinically with a variety of antigens. The advantage of SLA archaeosomes is that they exhibit a favorable safety profile and the inherent capability of stimulating both the humoral and cellular arms of the adaptive immune response system^3–8^.

Many enveloped viruses require surface glycoproteins for interaction with target cell receptors, thus making them ideal vaccine antigens to provoke virus-neutralizing humoral immune responses. Viral glycoproteins are particularly rich in N-linked glycans, which not only directly impact recognition by the host immune system, but which are also crucial for their proper trafficking, functionality and conformation^9,10^. In commercial SUVs, antigens are most commonly produced using yeast, insect, or more recently CHO cells. These cell types differ significantly in how they post-translationally modify recombinant proteins, especially N-linked glycans. Yeast and insect cells produce primarily mannose-rich (high- and/or pauci-mannose) glycans while CHO cells generate human-like, complex-type (CT) glycans. Mannose-rich glycans can also be produced in CHO cells, but these are typically further processed into complex forms by mannosidase I, which is followed by the addition of other types of sugars (GlcNAc, Gal and Neu5Ac)^11^.

For SARS-CoV-2, recombinant forms of the spike glycoprotein, produced in both insect and CHO cells, have been used as SUV antigens for clinical applications. SARS-CoV-2 SUVs using CHO-derived antigens have been approved by regulators globally^12^, while in North America and Europe, two of the approved SUVs, Nuvaxovid (Novavax) and Vidprevtyn Beta (Sanofi/GSK), are both produced in insect cells. Analyses of glycans present at the N-linked glycosylation sites of the SARS-CoV-2 spike protein produced in insect cells confirmed the presence of exclusively mannose-rich glycans^13,14^ whereas those produced in CHO cells predominantly contain complex glycans, which closely resembles the glycosylation profile expected of human-derived glycoproteins^15,16^. Despite the demonstrated efficacy and widespread clinical use of vaccines based on CHO and insect cell produced spike antigens, the impact of these distinct N-glycans profiles on the antigen’s immunogenicity has yet to be fully elucidated.

Given the abundance and important functions of glycans on viral glycoproteins, it may be expected that proteins modified with mannose-rich vs complex glycans would perform differently as vaccine antigens. Indeed, this has been tested for different enveloped viruses including HIV-1 gp120 (produced in 293 or Sf9 cells, adjuvanted with Freund’s Complete Adjuvant (FCA) or CpG and administered subcutaneously to BALB/c mice)^17^, HCV Env (produced in 293 or Sf9 cells, adjuvanted with Sigma adjuvant system (SAS) and administered intraperitoneally to CD-1 mice)^18^ and influenza A H5 (produced in 293, adjuvanted with Stimune and administered intramuscularly to C57BL/6 mice)^19^, although, the results of these tests for the different viruses/surface glycoproteins have not been consistent. For example, the high-mannose or paucimannose glycoforms found on the HIV-1 Env (gp120), HCV Env (E2) and influenza A hemagglutinin (H5) viral surface glycoproteins were reported to be more immunogenic, similarly immunogenic and less immunogenic, respectively, compared to the same proteins with CT glycosylation^17–19^. These immunological differences may be related to the number of N-linked glycosylation sites on these proteins, which ranged from 23 for gp120, 11 for E2 and 6 for H5 in the aforementioned studies.

For the SARS-CoV-2 spike, which has a similar number of glycosylation sites to HIV-1 gp120, HM glycoforms of spike protein generated using HEK293S N-acetylglucosaminyl transferase I (293GnT1)-deficient cells have been reported to be equivalent or better than their CT counterparts at inducing neutralizing antibody titers when administered intramuscularly to BALB/c mice^20,21^. However, this spike protein was shown to have significantly different biophysical properties than the same protein produced in GnT1-positive HEK293F cells, including markedly different thermal stability^21^. Notably, such changes have not been observed by our group for HM spike proteins produced in CHO cells (Supplementary Fig. S1), suggesting that host cell-dependent differences in protein structure may not be solely due to differences in the glycosylation profile. Furthermore, given the much more widespread use of CHO than HEK293 cells for therapeutic protein manufacturing, evaluation of the glycosylation-mediated effects on immunogenicity of CHO-derived antigens is merited.

Previously, we demonstrated that a uniformly HM glycoform (predominantly Man9) of recombinant SARS-CoV-2 spike protein can be effectively produced by supplementing CHO production cultures with kifunensine, a potent inhibitor of mannosidase I^16^. In the current study, we have compared this HM spike to the same protein produced in the absence of kifunensine (bearing human-like, predominantly complex-type, N-glycans) in SLA archaeosome-adjuvanted vaccine formulations in mice, monitoring both humoral and cellular immune responses. Compared to spike produced in the absence of kifunensine, sera from mice vaccinated with HM spike formulations exhibit significantly lower levels of neutralization activity, which is reflected by a spike subdomain-specific re-focusing of the humoral immune response towards subunit 2 (S2). We also demonstrate that a similar poorly-neutralizing, S2-dominant profile is observed with serum from mice immunized with SARS-CoV-2 spike protein produced in insect cells. These results indicate that the presence of CT N-glycans may be critical for directing the immune response towards neutralizing epitopes and could guide the choice of production hosts for ongoing development of new vaccines.

## Materials and Methods

### Antigens

SmT1 and SmT2 constructs are SARS-CoV-2 spike trimers produced in CHO^55E1^ cells as described previously^22,23^. Briefly, the SARS-CoV-2 reference strain spike ectodomain sequence (amino acids 1-1208 derived from Genbank accession number MN908947) was codon-optimized for CHO cells and synthesized by GenScript. Within the construct, the spike glycoprotein was preceded by its natural N-terminal signal peptide and fused at the C-terminus to human resistin (accession number NP_001180303.1, amino acids 23-108) and purification tags FLAG-(His)6 for SmT1 or fused at its C-terminus to the T4 phage foldon sequence and purification tags Flag-dual-Strep-(His)6 for SmT2. Mutations were added to stabilize the generated spike protein as previously described; amino acids 682-685 (RRAR) and 986-987 (KV) were replaced with GGAS and PP, respectively^24,25^. Constructs were cloned into the pTT5™ plasmid. Supernatants of transiently transfected CHO^55E1^ cells were harvested at 7 days post-transfection as described^22^.

SmT1 and SmT2 proteins were purified, respectively, using AVIPure-COV2S resin (Avitide, Lebanon, New Hampshire) or NGL COVID-19 (Repligen, Waltham, Massachusetts) spike affinity resins, according to the manufacturers’ instructions. Briefly, 0.2 μm-filtered supernatant was loaded on columns equilibrated with Dulbecco’s Phosphate Buffered Saline (DPBS). Columns were washed once with 5-10 column volumes of DPBS and protein eluted with 50 mM Bis-Tris, 1 M arginine-HCl, pH 6.0 (AVIPure-COV2S) or 100 mM sodium acetate, pH 3.5 (NGL COVID-19). The fractions containing eluted proteins were pooled and buffer exchanged for DPBS using desalting columns (GE Healthcare). Purified protein was quantified by spectrophotometry (A280) using extinction coefficient calculated based on the amino acid composition. Purified proteins were analyzed by SDS-PAGE using NuPAGE 4-12% Bis-Tris gels (Invitrogen) followed by Coomassie Blue staining. The absence of endotoxin contamination was verified using Endosafe cartridge-based Limulus amebocyte lysate tests (Charles River Laboratories, Charleston, SC, USA).

Production and evaluation of glycosylation for the baculovirus/Sf9-generated SARS-CoV-2 spike, referred to herein as Sm-gp41, was described previously^14^.

The S1, S2, NTD and RBD spike protein fragments used for ELISAs were produced and purified as described previously^7^.

Peptide mass spectrometry was used to confirm the identity and purity of all antigens tested.

### High-Performance Anion-Exchange Chromatography with Pulsed Amperometric Detection (HPAEC-PAD) measurements

#### Neutral sugar analysis

The dried protein sample (25 μg) was heated, in triplicate with TFA (160 μL, 5 M) (Sigma, cat# 91707) in an oven at 100 °C for 2 h. The sample was allowed to cool and then was evaporated to dryness (Genevac EZ-2 Elite). The sample was then re-suspended in water (125 μL), centrifuged at 14,000 rpm for 2 min, and then diluted with water 1:20 (v:v). 10 μl injections were eluted with a stepwise gradient of NaOH and were repeated in triplicate on Dionex ICS-6000 system equipped with a CarboPac PA20 column (3 × 150 mm, 060142), CarboPac PA20 guard column (3 × 30 mm, 060144), a BorateTrap column (4 × 50 mm, 047078) and a pulsed amperometric detector. Data collection and analysis have been performed with Chromeleon 7 (Thermofisher Scientific). The data was processed using GraphPad Prism 9.0.2.

### Surface plasmon resonance binding assays

Binding affinity (KD) of purified monomeric human ACE2 (hACE2) to the trimerized spike (S) proteins of SARS-CoV-2 was determined using a Biacore T200 surface plasmon resonance (SPR) instrument (Cytiva, Marlborough, MA). SPR experiments were carried out at 25 °C using PBS containing 0.05% Tween 20 (Teknova, Hollister, CA) with added 3.4 mM ethylenediaminetetraacetic acid (EDTA) as running buffer (PBST). Production and purification of recombinant hACE2 protein was carried out as described^23^. The binding analysis was performed in two steps, first using indirect capture of the S-protein trimer onto the SPR surface, followed by flowing the hACE2 or buffer blank over top to generate the binding sensorgrams. The SPR capture surface was generated using an anti-RBD single-domain antibody (V_H_H 11) fused to a human IgG1 Fc^26^. This antibody was diluted to 10 µg/mL in 10 mM sodium acetate immobilization buffer pH 4.5 (Cytiva, Marlborough, MA) and immobilized to approximately 2000 resonance units (RUs) onto a CM-5 sensor chip using the Immobilization Wizard for NHS/EDC amine coupling within the Biacore T200 Control instrument software (v2.0.1). The S-protein trimers under analysis were diluted to 10 µg/mL in PBST and captured onto the anti-spike surface at 10 µL/min for 180 s. The hACE2 – S-protein interactions were then assessed using single cycle kinetics analysis with three concentrations, using a 5-fold dilution from the top concentration of 200 nM. The hACE2 was injected at 50 µL/min over captured spike protein with a contact time of 150 s at each concentration and a 600 s dissociation. At the end of dissociation, the anti-spike surfaces were regenerated with a 30 s pulse of 10 mM glycine pH 1.5 at 30 µL/min. Sensorgrams were double referenced to the blank anti-spike sensor surface and analyzed for binding affinity and kinetics using the 1:1 binding model in the Biacore T200 Evaluation software (v3.0.2).

### Institutional review board statement, mouse immunization and sample collection

Mice were randomly assigned to groups and maintained at the small animal facility of the National Research Council Canada (NRC) following a 1 week acclimatization period in accordance with the guidelines of the Canadian Council on Animal Care. All procedures performed on animals in this study were approved by our Institutional Review Board (NRC Human Health Therapeutics Animal Care Committee) and covered under animal use protocols 2020.10. All experiments were carried out in accordance with the ARRIVE guidelines.

Female C57BL/6 mice (6–8 weeks old) were obtained from Charles River Laboratories (Saint-Constant, Canada). Based on the variability seen in the measured immune responses in previous studies, mouse experiments were conducted with *n* = 10 per group. Antigen and adjuvant vaccine components were admixed and diluted in PBS (Spectrum, Gardena, CA, USA) prior to administration in a final volume of 50 µL per dose. SLA archaeosomes are proprietary NRC adjuvants that were prepared, as previously described^27,28^. Levels of endotoxin in the SLA archaeosomes were verified by the Endosafe cartridge-based Limulus amebocyte lysate test (Charles River Laboratories) and confirmed to be <0.1 EU per mg.

Animals were immunized by intramuscular (i.m.) injection (50 µL) into the left tibialis anterior muscle on days 0 and 21 with various vaccine formulations as described above. On day 28, mice were anesthetized with isoflurane and then euthanized by cervical dislocation prior to collection of spleens for measurement of cellular immune responses by IFN-γ ELISpot. Mice were bled via the submandibular vein on day 28 with recovered serum used for quantification of antigen-specific IgG antibody levels and neutralization assays. Samples were simultaneously collected from 10 naïve animals for the assessment of background immune responses. Each of the samples from the individual mice was tested separately in the various readouts. No adverse events or humane endpoints were observed.

### Anti-spike IgG ELISA

Anti-spike total IgG titers in serum were measured by indirect ELISA with SmT1 (CT), SmT1 (HM) or fragments of SmT1 (CT), as previously described, except the blocking step was conducted with 2% bovine serum albumin^7,29^. Briefly, 96–well high-binding ELISA plates (Thermo Fisher Scientific) were coated with 0.3 µg/mL SmT1 protein diluted in PBS. Prior to adding serum and between each subsequent step the plates were washed with DPBS containing 0.05% (v/v) Tween 20 (Sigma-Aldrich, St. Louis, MO, USA). Serum samples were serially diluted 3.162-fold and added to the plates to allow for binding of antibodies to the protein. Bound IgG was detected with goat anti-mouse IgG -HRP (1:4000, Southern Biotech, Birmingham, AL, USA) prior to the addition of the substrate o-phenylenediamine dihydrochloride (Sigma-Aldrich). Bound IgG Abs were detected spectrophotometrically at 450 nm. Titers for IgG in serum were defined as the dilution that resulted in an absorbance value (A_450_) of 0.2 and was calculated using XLfit software (ID Business Solutions, Guildford, UK). Samples that did not reach the target OD were assigned the value of the lowest tested dilution (i.e., 100) for analysis purposes.

### IFN- γ ELISpot

IFN- γ ELISpot was also conducted as previously described^7,30^. The levels of spike glycoprotein specific T cells were quantified by ELISpot using a mouse IFN-γ kit (Mabtech Inc., Cincinnati, OH, USA). A spike peptide library (JPT Peptide Technologies GmbH) based on the reference strain sequence and consisting of 315 peptides (15-mers overlapping by 11 amino acids with last peptide consisting of a 17-mer) was used to stimulate splenocytes isolated from each of the mice. The library was split into three subpools and used to separately stimulate 4 × 10^5^ cells in duplicate at a final concentration of 2 µg/mL per peptide. Cells were also incubated without any stimulants to measure background responses. Spots were counted using an automated ELISpot plate reader (Cellular Technology LTD, Beachwood, OH, USA). For each animal, values obtained with media alone were subtracted from those obtained with each of the spike peptide pools, and then combined to yield an overall number of antigen-specific IFN-γ + SFC/10^6^ splenocytes per animal.

### Cell-based SARS-CoV-2 spike-hACE2 binding assay

The ability serum to neutralize the binding of labeled SARS-CoV-2 spike trimers (SmT1) to hACE2-expressing cells was performed similarly as previously described, using HEK293T-hACE2 cells (BEI Resources NR-52511)^7,31^. Indicated dilutions of mouse serum were mixed with 250 ng of biotinylated spike and 1 × 10^5^ HEK-293T-hACE2 cells. The amount of bound spike was quantified using Streptavidin-phycoerythrin conjugate by acquiring cells on an LSR Fortessa (Becton Dickinson) and analyzing data on FlowJo (Becton Dickinson). For illustration/analysis purposes, samples with calculated values ≤ 0 were assigned a value of 0.

### Pseudovirus neutralization assay

Pseudovirus neutralization assay was performed in 384-well plate format adapted from previously described protocol and modification^32,33^. Briefly, 4-fold serial dilutions of the serum samples were incubated with diluted virus at a 1:1 ratio for 1 h at 37 °C before addition to HEK293-hACE2/TMPRSS2 cells obtained from BEI Resources repository of ATCC and the NIH (NR-55293). Infectivity was then measured by luminescence readout per well. Bright-Glo luciferase reagent (Promega, E2620) was added to wells for 2 min before reading with a PerkinElmer Envision instrument. Neutralization Titer 50 (NT50) were calculated with nonlinear regression (log[inhibitor] versus normalized response – variable slope) with the 100% and 0% constraint. Pseudotyped lentiviral particles were produced expressing the SARS-CoV-2 variant spikes under CMV promotor and were packaged onto lentiviral vectors obtained through BEI Resources, NIAID, NIH: SARS-Related Coronavirus 2, Wuhan-Hu-1 (GenBank # NC_045512) spike-Pseudotyped Lentiviral Kit, NR-52948.

### ELISA-based epitope accessibility probing

Recombinant spike proteins variants were passively adsorbed onto NUNC® Immulon 4 HBX microtiter plates (Thermo Fisher, Ottawa, ON, Canada) at 50 ng/well in 100 µL of PBS, overnight at 4 °C. The following day, plates were blocked with PBSC (PBS with 1% [w/v] casein [Sigma, Oakville, ON, Canada]) for 1 h at room temperature. The panel of V_H_H-Fcs were then added at 1 µg/mL in PBST (PBS supplemented with 0.05% [v/v] Tween® 20) and incubated for 1 h at room temperature. After this, the plates were washed five times with PBST and incubated with 1 µg/mL of HRP-conjugated goat anti-human IgG (Sigma, Oakville, ON, Canada) for 1 h. Following an additional five washes with PBST, 100 µL of peroxidase substrate solution (SeraCare, Milford MA, USA) was added and incubated for 15 min at room temperature. The reaction was stopped by adding 50 µL/well of 1 N H_2_SO_4_, and the absorbance was measured at 450 nm using a Multiskan™ FC photometer (Thermo Fisher Scientific). Assay was performed in duplicate. Data were graphed using GraphPad Prism version 10.1.2 for Windows (GraphPad Software).

### Statistics and reproducibility

Data were analyzed using GraphPad Prism version 10 (GraphPad Software). Statistical significance of the difference between groups was calculated by two-tailed unpaired t test with Welch’s correction or one-way ANOVA followed by Tukey’s multiple comparisons test. Data were log-transformed (except for data from surrogate cell-based spike-hACE2 binding assay) prior to statistical analysis. For all analyses, differences were considered to be not significant with *p* > 0.05. Significance was indicated in the graphs as follows: *p < 0.05, **p < 0.01, ***p < 0.001, and ****p < 0.0001. Mouse experiments were conducted with 10 biological replicates (*n* = 10 mice per group) to maximize reliability. Initial allocation of animals into each group was done in a blinded fashion, afterwards the study proceeded in an unblinded fashion. No samples were excluded from the analysis.

### Reporting summary

Further information on research design is available in the Nature Portfolio Reporting Summary linked to this article.

## Results

### Antigen characterization

**Fig. 1:**
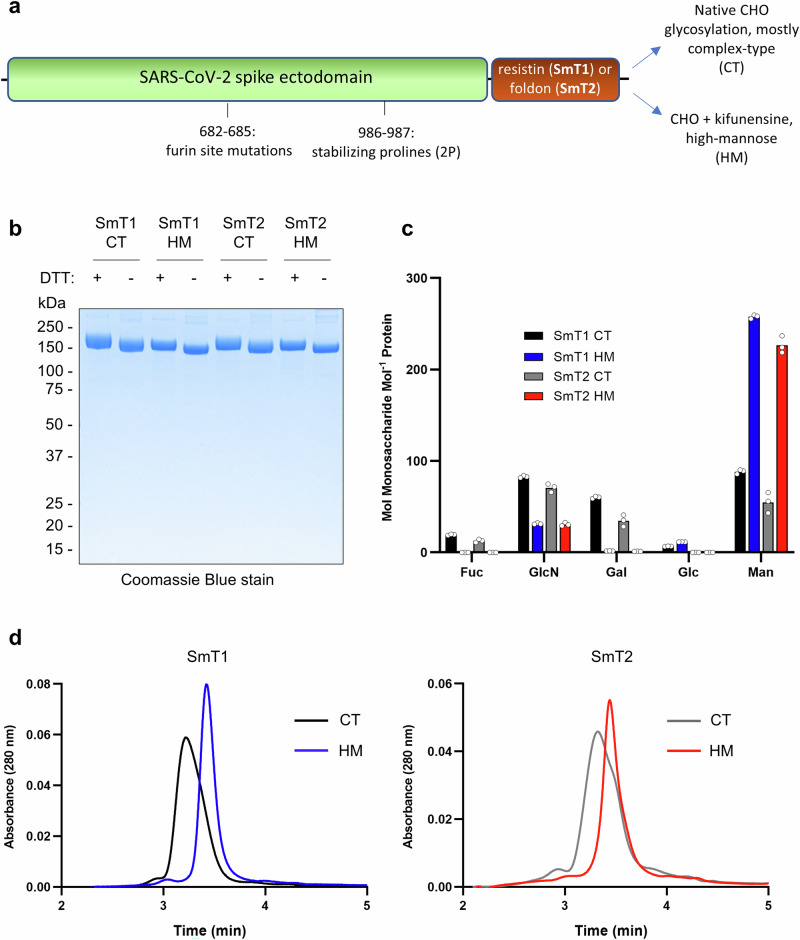
Addition of kifunensine during production in CHO cells affects spike trimer glycosylation. **a** Schematic of construct design for recombinant SARS-CoV-2 spike proteins used in the current study. **b** Reduced ( + DTT) and non-reduced (-DTT) SDS-PAGE analysis, vertically separated numbers represent ladder separation in kDa, **c** monosaccharide analysis (HPAEC-PAD; mean of *n* = 3 technical replicates shown by open circles), and **d** analytical size exclusion chromatography (representative of multiple runs).

Recombinant trimeric SARS-CoV-2 spike proteins SmT1 and SmT2 were produced in CHO cells as described previously^7,22^. As shown in Fig. 1a, both constructs consist of the full spike ectodomain (reference Wuhan-Hu-1 sequence) with prefusion-stabilizing ‘2 P’ and furin site mutations but with distinct C-terminal trimerization sequences: human resistin for SmT1 and T4 bacteriophage foldon for SmT2. We have performed extensive biophysical characterization of SmT1 and SmT2 previously, and resistin and foldon were similarly effective at mediating spike trimerization^34^. To compare the immunogenicity of CT and HM glycoforms of these spike antigens, proteins were first produced in CHO cultures in the absence or presence of kifunensine, respectively. CT and HM forms of SmT1 and SmT2 were purified by spike-affinity chromatography, giving excellent purity as assessed by Coomassie-stained SDS-PAGE (Fig. 1b). Notably, kifunensine treatment does not significantly affect the SDS-PAGE migration pattern of SmT1 or SmT2.

Detailed analysis of glycosylation of SmT1 produced in the presence or absence of kifunensine was reported by our group in a recent publication, which included LC-MS glycopeptide analysis, hydrophilic interaction chromatography with fluorescence detection (HILIC-Fld) and high-performance anion-exchange chromatography with pulsed amperometric detection (HPAEC-PAD) monosaccharide analysis^16^. Compared to the mostly CT glycans formed without kifunensine treatment, the HM form contains almost undetectable CT glycans with high-mannose structures, specifically Man9, predominating. Correlating with the near absence of CT glycans, SmT1 contains very low levels of sialic acid^16^. In the current study, we performed HPAEC-PAD analysis of the HM and CT glycoforms of SmT1 and SmT2 with TFA hydrolysis to quantify fucose (Fuc), glucosamine (GlcN), glucose (Glc), galactose (Gal) and mannose (Man) sugars, but we did not repeat the sialic acid, LC-MS or HILIC-Fld analyses performed previously^16^. As shown in Fig. 1c, GlcN (derived from N-acetylglucosamine during hydrolysis) levels are reduced and Gal is nearly absent in SmT1 and SmT2 produced with kifunensine while Man levels are substantially increased (consistent with inhibition of mannosidase I enzyme and formation of primarily HM glycans) compared to the same proteins produced without kifunensine. These results are consistent with the previous study and confirm that high-mannose forms of SmT1 and SmT2 were successfully produced.

By size-exclusion chromatography (SEC; Fig. 1d), SmT1 and SmT2 produced without kifunensine show a single predominant elution peak, as observed previously, which corresponds to a trimeric species^34^. Low levels of faster-eluting hexameric species and slower-eluting low molecular weight species are also detected. When produced with kifunensine supplementation, the primary elution peaks for SmT1 and SmT2 are significantly narrower, possibly reflecting reduced size heterogeneity due to a more uniform HM glycosylation. In addition, and as already reported elsewhere^20^, elution of the kifunensine-treated proteins from the SEC column is slightly delayed. Based on multi-angle light scattering (MALS) analysis of the SEC peaks, the trimeric forms of SmT2 produced with and without kifunensine are estimated to have molecular weights (including glycans) of 530 and 540 kDa, respectively. This small difference in molecular weight likely does not explain the shift in SEC elution times of the two species; it is more likely due to another factor such as increased matrix interactions for the HM glycoform in the absence of the charged glycans (e.g., neuraminic acid) normally present in the spike protein, or to a more compact quaternary structure.

### Comparison of spike glycoforms as vaccine antigens in mice

To evaluate the immunogenicity of these spike glycoforms as vaccine antigens, separate studies were conducted with the resistin-trimerized SmT1 or T4 foldon-trimerized SmT2. Mice (*n* = 10 per group) were immunized at days 0 and 21 with vaccines formulated with 1 µg of each of the aforementioned recombinant proteins adjuvanted with SLA archaeosomes. Antigen-specific humoral and cellular responses were evaluated by ELISA, neutralization assay and IFN-γ ELISpot.

### Immunogenicity of SmT1 CT and HM glycoforms

**Fig. 2:**
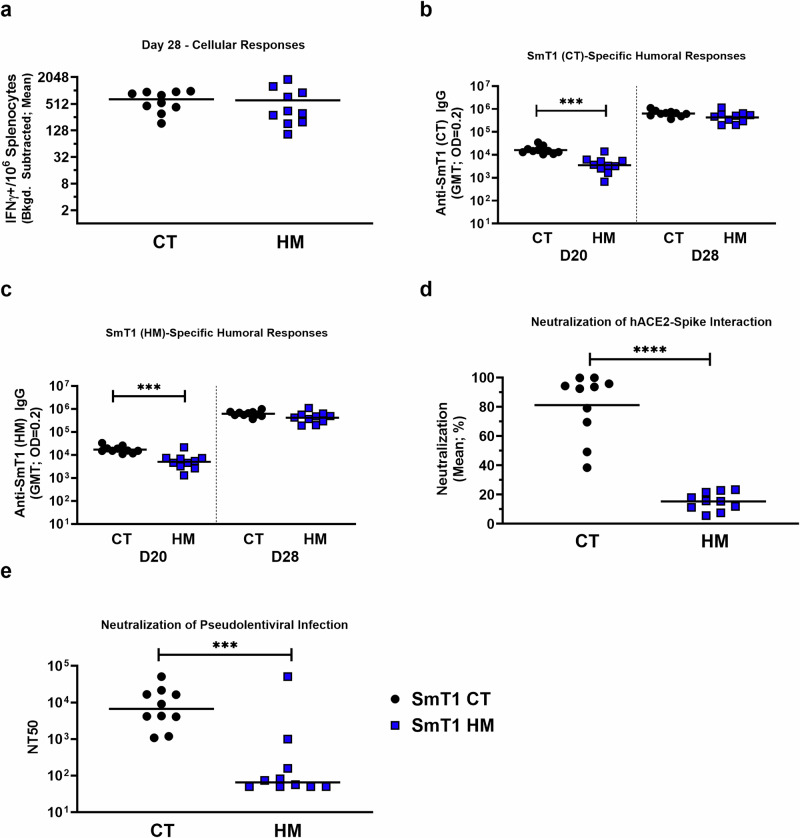
SLA archaeosome-adjuvanted resistin-trimerized SARS-CoV-2 spike antigen induces enhanced neutralizing responses with complex type (CT) compared to high mannose (HM) N-linked glycans. C57BL/6 mice (*n* = 10/group) were immunized i.m. with SmT1, either CT or HM, adjuvanted with SLA on days 0 and 21. **a** Splenocytes were harvested on Day 28 and analyzed by IFN-γ ELISpot when stimulated by spike peptide pools. Values obtained with media alone were subtracted from those measured in the presence of the peptides. Serum was collected and analyzed by ELISA against (**b**) SmT1 (CT) or (**c**) SmT1 (HM) to determine the antibody titers on Day 20 and Day 28. Serum was collected on Day 28 and analyzed for neutralizing activity against reference SARS-CoV-2 spike with two methods. Blocking of the spike-hACE2 interaction (**d**) was assessed in a surrogate cell-based SARS-CoV-2 spike-hACE2 binding assay. In brief, labeled recombinant spike trimers, mouse serum (diluted 1 in 75) and hACE2-expressing cells were co-incubated and the spike-hACE2 interactions were quantified by flow cytometry. In a similar fashion, blocking of a productive infection (**e**) was assessed by a pseudolentiviral neutralization assay. In brief, SARS-CoV-2 spike pseudotyped lentivirus, mouse serum (4-fold serial dilutions) and hACE2/TMPRSS2 expressing cells were co-incubated and productive infection was quantified by luciferase assay to determine the Neutralizing Titer 50 (*NT50*). In all graphs, statistical significance of differences is shown as: ***p < 0.001 and ****p < 0.0001 by two-tailed unpaired t test with Welch’s correction when comparing CT and HM groups. *IFN*γ*:* Interferon gamma; *Bkgd:* background; *CT:* Complex-type; *HM****:*** High mannose; *PM:* Paucimannose; *OD:* Optical Density; *GMT:* Geometric Mean Titer.

Cellular responses induced by the two glycoforms of SmT1 were evaluated by IFN-γ ELISpot using splenocytes collected on Day 28 with a peptide pool encompassing the entire length of the SARS-CoV-2 reference-strain spike protein as a stimulant (Fig. 2a). The two groups of vaccinated mice showed no significant difference in levels of spot forming cells (SFCs), regardless of glycoform used (mean of 648 vs 616 with CT and HM antigens, respectively). The humoral response was also assessed. SmT1 (CT)-specific IgG was evaluated 1 day prior to boosting (Day 20) and 7 days post-boost (Day 28) by ELISA (Fig. 2b). In Day 20 serum, SmT1 CT vaccine recipients exhibited a small but significant increase in spike-specific IgG compared to SmT1 HM-vaccinated mice (Geometric Mean Titer (GMT) 16,074 vs 3,508; *p* < 0.001). Post-boost, differences in SmT1 (CT)-specific IgG was no longer significant, although there was still a trend towards lower titers in the HM-immunized group (GMT 636,684 vs 431,350; *p* = 0.0687). To account for potential bias in glycosylation-specific IgG reactivity based on the CT glycoform coating antigen used, the ELISA was repeated using plates coated with the HM glycoform of SmT1 (Fig. 2c). As before, SmT1 CT-vaccinated mice had a significantly higher spike-specific IgG detected at Day 20 (GMT 17,175 vs 5,017; *p* < 0.001), whereas there was no significant difference post-boost, at Day 28 (GMT 617,785 vs 416,604; *p* = 0.0583). Thus, levels of anti-spike IgG were consistent regardless of the glycoform used for the ELISA procedure.

To assess the functionality of the spike-specific IgGs induced by the two SmT1 glycoforms, a cell-based SARS-CoV-2 spike-hACE2 binding assay was performed as a surrogate neutralization assay using post-boost (Day 28) serum (Fig. 2d). Interestingly, when adjuvanted with SLA, the CT glycoform was able to elicit a much stronger neutralizing antibody response when compared to the HM glycoform (mean of 81% vs 15%; *p* < 0.0001). Consistent with our previous reports^31,35^, the trends observed in the surrogate neutralization assay were confirmed with an orthogonal pseudolentiviral neutralization assay, which evaluates the ability of serum to neutralize infection of spike-pseudotyped lentivirus to cells expressing hACE2 (Fig. 2e). Here, the serum from SmT1-CT vaccine recipients exhibited 2 orders of magnitude more neutralizing activity compared to serum from SmT1-HM vaccinated mice (median of 6,681 vs 65; *p* < 0.001). This implies that although total anti-spike IgG is similar for groups immunized with the two glycoforms, the quality of the response, in terms of induction of spike-neutralizing antibodies, is greatly improved when the CT antigen is used.

### Immunogenicity of CT, HM and PM glycosylated spike

In previous studies, our group has validated extensively the effectiveness of resistin-trimerized spike as a vaccine antigen against SARS-CoV-2 in animal models. Nonetheless, the bacteriophage T4 foldon has been a more widely used trimerization domain for spike structural studies and for vaccine antigens; notably, a spike-foldon fusion serves as the antigen (insect cell-derived) in Sanofi’s COVID-19 vaccine (Vidprevtyn beta) which has achieved regulatory approval in Europe.

**Fig. 3:**
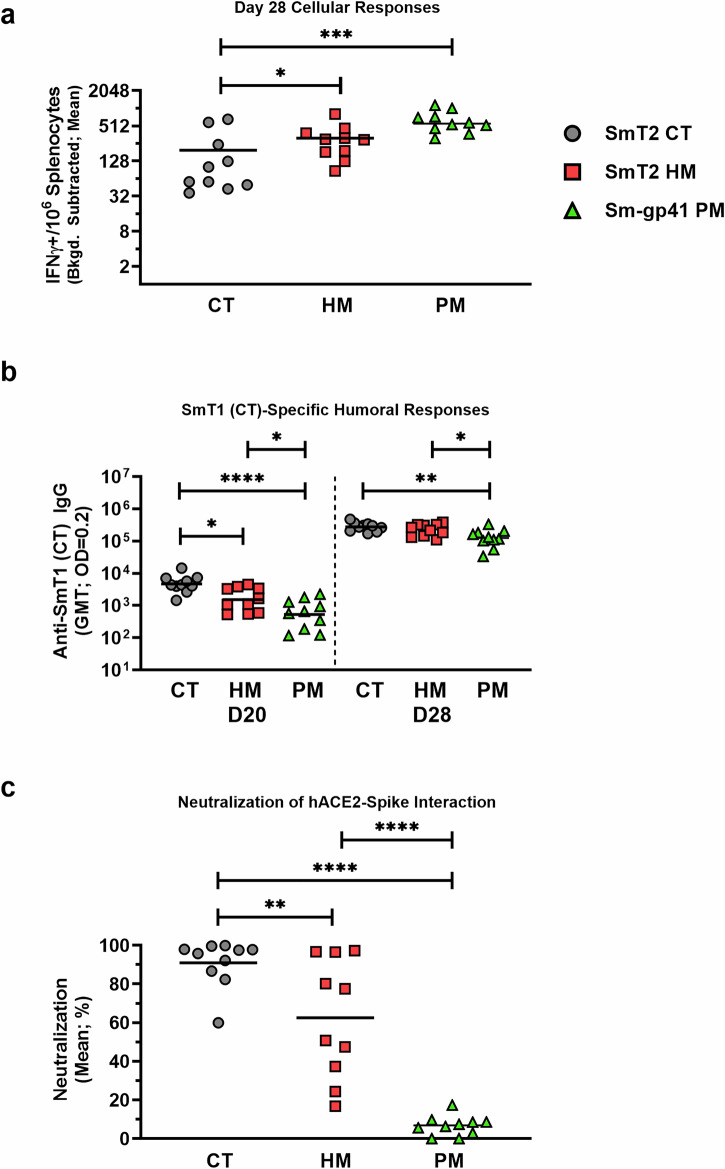
SLA archaeosome-adjuvanted T4 foldon-trimerized SARS-CoV-2 spike with complex type (CT) N-linked glycans induces enhanced neutralizing responses compared to vaccines containing spike with high mannose (HM) or paucimannose (PM) glycans. C57BL/6 mice (n  =  10/group) were immunized i.m. with SmT2, either CT or HM, or Sm-gp41 (PM), adjuvanted with SLA archaeosomes on days 0 and 21. **a** Splenocytes were harvested on Day 28 and analyzed by IFN-γ ELISpot when stimulated by spike peptide pools. Values obtained with media alone were subtracted from those measured in the presence of the peptides. **b** Serum was collected and analyzed by ELISA against SmT1 (CT) to determine the antibody titers on Day 20 and Day 28. **c** Serum was collected on Day 28 and analyzed for neutralizing activity against reference SARS-CoV-2 spike in a surrogate cell-based SARS-CoV-2 spike-hACE2 binding assay. In brief, labeled recombinant spike trimers, mouse serum (diluted 1 in 75) and hACE2-expressing cells were co-incubated and the spike-hACE2 interactions were quantified by flow cytometry. In all graphs, statistical significance of differences is shown as: *p < 0.05, **p < 0.01, ***p < 0.001 and ****p < 0.0001 by one-way ANOVA followed by Tukey’s multiple comparisons test. *IFN*γ*:* Interferon gamma; *Bkgd:* background; *CT:* Complex-type; *HM:* High mannose; *PM:* Paucimannose; *GMT:* Geometric Mean Titer; *OD:* Optical Density.

To confirm that the observed effect of glycosylation status on spike immunogenicity was not limited to spike-resistin fusions, a separate study was conducted with CT and HM glycoforms of T4 foldon-trimerized SARS-CoV-2 spike (SmT2). These antigens were produced using the same CHO^55E1^ expression platform and mice were vaccinated as before. As shown in Fig. 3a, by IFN-γ ELISpot, the SmT2 HM-formulated vaccine elicited a slightly stronger (*p* < 0.05) cellular response than the SmT2 CT vaccine: mean IFN-γ SFCs for the HM and CT groups were 316 and 196, respectively. The differences in humoral responses between the SmT2 CT and HM groups were very similar to those observed for SmT1: in pre-boost (Day 20) samples, SmT2 CT vaccine recipients exhibited a small but significant increase in total spike-specific IgG compared to the SmT2 HM group (GMT 4,696 vs 1,520; p < 0.01), a difference which was no longer significant at Day 28, 7 days post-boost (Fig. 3b). For SmT2, the HM glycoform induced significantly lower levels of spike-neutralizing antibodies compared to the CT glycoform (mean of 91% for CT and 63% for HM; Fig. 3c), although the magnitude of the difference was considerably less than for the two glycoforms of SmT1 (Fig. 2). This result suggests that the impact of glycosylation on SARS-CoV-2 spike immunogenicity is in part dependent on the identity of the fusion partner chosen to mediate trimerization; nonetheless, compared to paired CT controls for SmT1 and SmT2, the HM antigens consistently evoked lower levels of neutralizing antibodies, despite slightly better induction of cellular immunity.

In the experiment performed with the two SmT2 glycoforms (Fig. 3), an additional group of mice was immunized with an SLA-adjuvanted formulation containing soluble spike trimers produced in Sf9 insect cells, Sm-gp41, a paucimannose (PM) glycoform of SARS-CoV-2 spike^14^. Previously published analysis of N-linked glycosylation showed that HM and PM glycans predominate in this construct, as expected for an insect cell-derived glycoprotein. Notably, although this construct consists of the same reference-strain SARS-CoV-2 spike sequence as SmT1 and SmT2, a distinct fusion partner, the HIV gp41 molecular clamp^36^, is used to mediate trimerization. Interestingly, the IFN-γ ELISpot (Fig. 3a) indicates that the induction of cellular immunity is significantly higher for the PM spike, compared to SmT2 CT (640 vs 196; *p* < 0.01). The opposite trends are observed when evaluating humoral immunity (Fig. 3b), as total anti-spike IgG elicited by vaccines based on the PM spike remained significantly lower than those based on the CT spike, at both Day 20 (824 vs 5,560; *p* < 0.0001) and Day 28 (143,356 vs 289,481; *p* < 0.01). Still, it is surprising that the post-boost serum neutralization activity (Fig. 3c) is profoundly reduced in the group vaccinated with the PM spike compared to the CT spike (7% vs 91%; *p* < 0.0001). Importantly, this effect could be explained by differences in glycosylation or by the different trimerization sequence of Sm-gp41 compared to SmT2 (or by a combination of these factors).

### Mechanisms by which glycosylation impacts spike immunogenicity

**Fig. 4:**
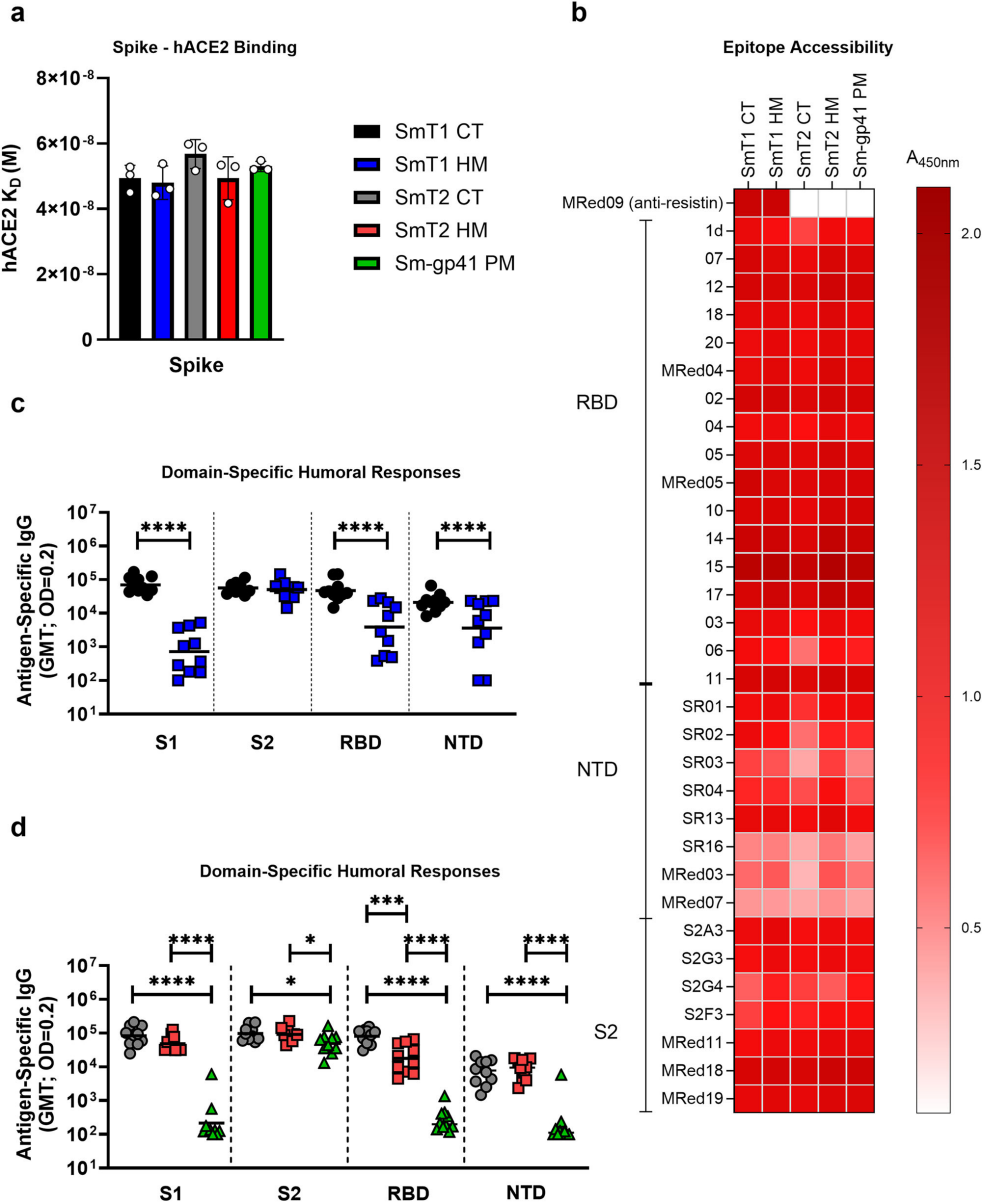
Presence of complex-type N-linked glycans on SARS-CoV-2 spike enhances the immunogenicity of the receptor binding domain (RBD), without impacting receptor affinity or epitope accessibility. **a** SPR determination of equilibrium dissociation constants (*K*_D_) for hACE2 binding (mean with SD of *n* = 3 injection cycles with independent hACE2 dilutions and captured S protein surfaces; values for individual injection cycles are shown by open circles). **b** Anti-spike V_H_H binding analysis against purified recombinant SARS-CoV-2 spike proteins. These VHH were previously characterized to bind regions within the corresponding to NTD (amino acids 16-305), RBD (amino acids 319-541) and S2 (amino acids 686-1208)^26^. **c**, **d** Day 28 serum from two independent mouse studies where SmT1- (**c**) or SmT2- and Sm-gp41 (**d**) immunized animals were analyzed by ELISA against domains of SARS-CoV-2 spike. In all graphs, statistical significance of differences is shown as: *p < 0.05, **p < 0.01, ***p < 0.001 and ****p < 0.0001 by (**a**, **d**) one-way ANOVA followed by Tukey’s multiple comparisons test or (c) two-tailed unpaired t test with Welch’s correction. *CT:* Complex-type; *HM:* High mannose; *PM:* Paucimannose; *GMT:* Geometric Mean Titer; *OD:* Optical Density.

Different hypotheses were considered to explain the reduced capacity of HM spike-based, SLA-adjuvanted vaccine formulations to induce spike-neutralizing antibodies in mice. First, as there have been reports of glycosylation-dependent RBD functionality^10,37,38^, differences in conformation of the ACE2 binding site could explain the observed differences between glycoforms. However, as shown in Fig. 4a, equilibrium dissociation constants *K*_D_s for ACE2 binding, determined by SPR, were not significantly different for the CT and HM glycoforms of SmT1 and SmT2 or for Sm-gp41 (4.8–5.7 × 10^-8 ^M). Neutralizing antibodies may also have epitopes that do not include but are proximal to the receptor binding site; to evaluate whether the conformations of such epitopes could be affected by differences in glycosylation, we tested binding of the different spike constructs by ELISA to a large set of previously characterized anti-spike V_H_Hs, many of which are neutralizing and bind diverse epitopes within the RBD^26^. As described by Rossotti et al., these V_H_H were isolated from immunized llamas and their targets were identified by library panning and screening using SARS-CoV-2 spike fragments (corresponding to NTD (amino acids 16-305), RBD (amino acids 319-541) and S2 (amino acids 686-1208)); the precise epitopes were mapped and defined previously by hydrogen-deuterium exchange mass spectrometry (HDX-MS)^26^. As shown in Fig. 4b, the binding profile of the different antigens to the panel of V_H_Hs is highly similar. Several NTD- and S2-specific V_H_Hs were also included, and binding of these antibodies to the spike glycoforms was also generally similar. Together, these results indicate that the tested antigens are highly similar and that differences in immune reactivity or ACE2-binding functionality likely do not explain the differences in the immune responses they are able to provoke.

We proceeded to examine whether differences in glycosylation could alter the spike sub-domain specificity of antibodies induced by adjuvanted vaccine formulations. For this, ELISAs were performed using purified spike S1, S2, NTD and RBD subunits/domains as coating antigens. Remarkably, for the SmT1 glycoforms, post-boost sera from the CT and HM-vaccinated groups show markedly different levels of domain-specific antibodies (Fig. 4c) targeting the S1 (GMT of 79,715 vs 1,659; *p* < 0.0001), which includes epitopes within the RBD (GMT of 59,113 vs 10,137; *p* < 0.0001) and NTD (GMT of 24,610 vs 10,659; *p* < 0.0001). Interestingly, similar levels of S2-specific IgG (GMT of 58,901–60,279) were detected in both groups. Comparing SmT2 glycoforms (Fig. 4d), only RBD-specific antibodies are significantly different for the groups receiving CT vs HM antigen (GMT of 90,026 vs 26,426; *p* < 0.001). Finally, for the Sm-gp41 (PM) antigen, levels of domain-specific IgG for the S1, RBD and NTD were barely detectable in most animals, only S2-specific IgGs were induced to a similar order of magnitude to SmT2 (CT) (GMT of 60,644 vs 108,847; *p* < 0.05). Together, these results indicate that lower induction of neutralizing antibodies of HM and PM glycoforms is correlated with a shift in regional immunogenicity towards the spike S2 subunit.

## Discussion

The glycosylation of viral surface glycoproteins is intricately linked to their function and ability to evade the immune system. Glycans play essential roles for proper folding and receptor interactions; for SARS-CoV-2 spike, although less heavily glycosylated than the surface glycoproteins of other viruses such as HIV, over 40% of the ectodomain surface is still estimated to be covered with glycans. Glycan structure also directly impacts antigen immunogenicity due to inherent differences in immune activation properties^39^. There have been a number of studies that have demonstrated a variability in the immunogenicity of viral glycoprotein antigens with altered glycosylation states^17–21,39–44^.

Herein, we evaluated how CT and HM glycoforms of CHO cell-derived recombinant SARS-CoV-2 spike trimers compare as antigens in a mouse vaccination model. In the choice of adjuvant, we selected SLA archaeosomes, as it would allow us to look at the impact of glycosylation on both the cellular and humoral immune responses. In addition, we have extensive experience comparing its immunogenicity to clinically approved adjuvants^4^. Particularly for SARS-CoV-2 antigens, we have previously shown that the hierarchy of immunogenicity induced by multiple spike variants is similar whether using SLA archaeosomes or a mimetic of the oil-in-water emulsion adjuvant system (AS03)^31,35^. These data are directly in line with other studies that infer regional immunogenicity of an antigen is conferred by the antigen^17,21,45^. We found that HM and PM spike produced in CHO (via kifunensine supplementation) and in insect cells, respectively, significantly impaired its ability to induce spike-neutralizing antibodies when formulated in a vaccine adjuvanted with SLA archaeosomes. This was similar to a previous study comparing the immunogenicity of SARS-CoV-2 spike antigen generated in 293 F (CT glycosylation), GnT1-deficient 293S (HM glycosylation, mainly Man5) and insect cells (PM glycosylation)^21^. They utilized two separate adjuvant systems, aluminum salts and Freund’s complete adjuvant, which both illustrated that the ability to induce antigen-specific antibodies was markedly improved in the CT vs PM antigen, regardless of adjuvant choice^21^. Interestingly, in our study, this effect was accompanied by only minor differences in vaccine-induced total anti-spike IgG, and by a slight, but significantly increased cellular immunity, especially for the PM spike. Several potential explanations for the difference in neutralization responses to these two glycoforms can be given, however, based on our data, we conclude that HM and PM glycosylation leads to focusing of the humoral immune response towards the spike S2 subunit, thereby reducing S1-directed antibodies that are more likely to block the RBD-ACE2 interaction. Lin et al., have shown similar trends when evaluating the immunogenicity of influenza hemagglutinin-derived from CHO or insect cells^40^. When adjuvanted with a combination formulation of a water-in-oil emulsion and a TLR9 agonist, CpG oligonucleotide, the insect cell-generated antigen induced similar or higher antigen-specific antibody titers and cellular responses, but much weaker neutralization activity to various pseudotyped viruses. Overall, it appears that the improved ability of antigens with CT glycans to induce neutralizing antibody responses may not be specific to adjuvant or antigen. In future studies, to improve structural insights into the impacts of glycosylation on immunogenicity, precise epitope mapping on polyclonal animal serum should be conducted by methods such as HDX-MS^46^.

Based on the results of the current study, we can only speculate on the mechanisms behind this altered response. The observed increase in cellular immunity in this study correlated with reduced complexity of glycan structures. This may be explained through enhanced interactions of antigen with mannose receptors on antigen presenting cells, such as dendritic cells or macrophages, leading to improved MHC-presentation and T cell activation^47,48^. As for the glycosylation-dependent antibody response, one plausible explanation involves differences in the type and complexity of glycans present at different parts of the spike: the predominant glycans of the spike “stalk” (at the C-terminus of the S2 subunit) were reported to be more processed (tri- and tetra-antennary, complex type) than elsewhere in S2 or S1, which are a mix of smaller complex and high-mannose structures^49–51^. The large size of these S2 stalk glycans could indicate a greater importance in shielding this part of the spike from the immune system. Their replacement by HM or PM glycans could thus lead to immune-dominance and contribute to the re-focusing of the humoral immune response towards this region. Our group recently confirmed, in two independent studies^15,16^ the increased complexity of glycosylation occurring in the spike “stalk” in our CHO produced antigens. It will be important for future studies to evaluate the potential role of various immune cells and signaling pathways in determining the nature of the immune response induced by differently glycosylated antigens, with a focus on translatability between mice and humans.

There are several potential explanations for discrepancies between our results and those obtained previously with HM spike proteins derived from GnT1^-^ HEK293 cells^20,21^, including differences in vaccine formulation and protein dosing. Also, the mannosidase I enzyme inhibited by kifunensine acts prior to GnT1 during the process of mammalian N-linked glycan maturation, such that the predominant glycans produced with kifunensine supplementation are larger (Man9) compared to those from GnT1^-^ cells (Man5)^52^. Finally, as already mentioned, the thermal stability of GnT1^-^ HEK293-derived spike appears to be significantly different than HEK293-derived CT spike^21^. For CT and HM glycoforms of SmT1 produced in CHO cells, primary melting transitions occur at 45.97-45.98 °C regardless of glycosylation state (Supplementary Fig. S1). This suggests that the altered thermal stability of spike protein from GnT1^-^ HEK293 cells is either due to the differences in mannose chain lengths (Man5 vs Man9) or other undefined changes affecting protein structure beyond differences in glycosylation which may also affect its performance as a vaccine antigen.

For manufacturing of glycoprotein-based subunit vaccines, it would seem preferable to use a human host cell line, such as HEK293, in order to most closely match the glycosylation pattern of viral antigens produced during infection. However, we and others have shown that HEK293 cells are capable of producing only low yields of recombinant SARS-CoV-2 spike trimers, typically less than 50 mg/L^22^, while CHO cells can achieve yields >1 g/L^53,54^. Also, while a recent study has confirmed certain differences between the N-linked glycans of HEK293 and CHO cell-derived spike proteins^55^, among production host cell lines commonly used for manufacturing therapeutic recombinant proteins, CHO cells generate the most human-like N-linked glycans^56^. For other viruses, CHO cells are becoming a production host of choice for subunit vaccine manufacturing^12^, building on their longstanding use for large-scale commercial production of monoclonal antibodies and other recombinant protein therapeutics, whereas HEK293 cells are rarely used for these purposes. Therefore, our findings that CT glycosylation is important for subunit vaccine performance specifically in the context of CHO-derived product is of particular relevance for commercial vaccine production.

Insect cells are also used extensively for vaccine manufacturing including for clinically-proven COVID vaccines (e.g., Nuvaxovid and Vidprevtyn Beta). Therefore, it was surprising that the insect cell-derived spike that was tested in the current study was so ineffective at inducing neutralizing antibodies compared to the CHO-derived proteins, despite similar ACE2 and anti-spike V_H_H binding profiles. This difference can potentially be explained, at least in part, by a different trimerization sequence that was used for the insect cell-derived spike (gp41 molecular clamp)^36^. However, it is notable that the S2-shifted immune response observed for the CHO-derived HM spike proteins was also apparent for the insect cell-derived spike. Interestingly, the commercialized, insect cell-derived COVID vaccine from Sanofi, Vidprevtyn Beta, is based on a foldon-trimerized spike antigen. In our hands, foldon-fused spike from CHO cells (SmT2) was less sensitive to glycosylation-dependent effects than the resistin-fused spike we tested (SmT1). Furthermore, it has been shown that improved neutralizing responses can be elicited from insect cell-derived antigen by altering the choice and strength of adjuvant within the vaccine formulation^21^. Thus, the use of foldon for trimerization with an insect cell-derived antigen in combination with the proper adjuvant may mitigate possible negative effects of HM or PM glycosylation on the ability of a vaccine to induce spike-neutralizing antibodies.

Together, our results highlight that although the impacts are not always consistent for different types of virus antigens, differences in glycosylation, specifically complex- vs high-mannose types, can have dramatic effects on the ability of a surface glycoprotein-based subunit vaccines to induce a high-quality immune response capable of blocking virus-host cell receptor interactions. The expression platforms used for commercial subunit vaccine manufacturing are predominantly based on non-human hosts (i.e., mostly insect, yeast and nowadays CHO cells), which generate N-linked glycans with varying degrees of similarity to those produced by human cells. The choice of a production host is therefore critical and should be carefully evaluated to maximize the immunogenic potential of new subunit vaccine candidates. Future research should be directed towards understanding the mechanisms responsible for the glycosylation-dependent subdomain immune re-targeting that we have observed and described here. In addition, engineering of new production hosts should be pursued, for example to enable complex glycosylation in insect cells or to fine-tune CHO cells to produce recombinant protein antigens with tailored glycans, allowing the most effective immune responses to be induced by adjuvanted vaccine formulations.

## Supplementary information

Supplementary Information

Description of Additional Supplementary Data

Supplementary Data 1

reporting summary

## Data Availability

Source data underlying all figures can be found in Supplementary Data 1.
