# Supplementary material for: Modifying the glycosylation profile of SARS-CoV-2 spike-based subunit vaccines alters focusing of the humoral immune response in a mouse model
Source: Commun Med (Lond). 2025 Apr 11;5:111. doi: 10.1038/s43856-025-00830-w (PMC11992040; doi:10.1038/s43856-025-00830-w)
Supplement: Supplementary file 1 — Supplementary Information [file 43856_2025_830_MOESM1_ESM.pdf]

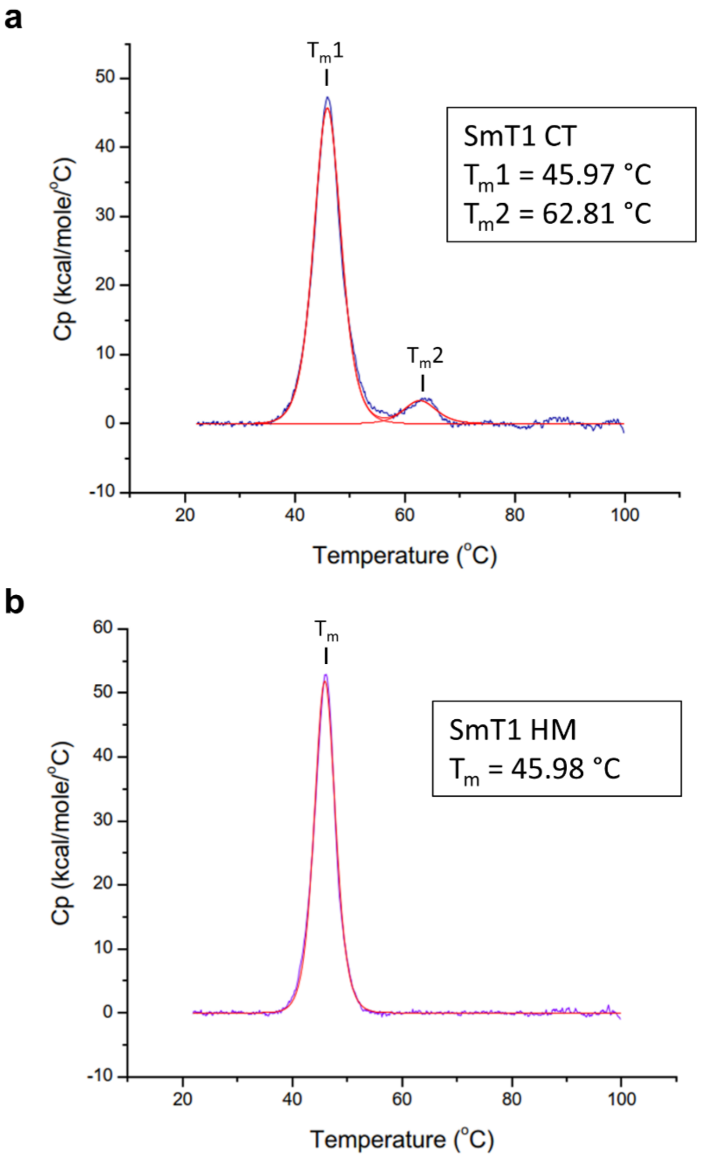

Supplementary Figure S1: SmT1 thermal stability is not impacted by simplifying the glycosylation state. Differential scanning calorimetry (DSC) was used to determine the thermal stability of SmT1 glycoform CT (a) and HM (b).
