# Supplementary material for: Modifying the glycosylation profile of SARS-CoV-2 spike-based subunit vaccines alters focusing of the humoral immune response in a mouse model
Source: Commun Med (Lond). 2025 Apr 11;5:111. doi: 10.1038/s43856-025-00830-w (PMC11992040; doi:10.1038/s43856-025-00830-w)
Supplement: Supplementary file 2 — Description of Additional Supplementary Data [file 43856_2025_830_MOESM2_ESM.docx]

**Description of Additional Supplementary Files**

**File Name**: Supplementary Data 1

**Description**: Source data for Figures 1-4 and Supplementary Figure 1
