# Supplementary material for: Modifying the glycosylation profile of SARS-CoV-2 spike-based subunit vaccines alters focusing of the humoral immune response in a mouse model
Source: Commun Med (Lond). 2025 Apr 11;5:111. doi: 10.1038/s43856-025-00830-w (PMC11992040; doi:10.1038/s43856-025-00830-w)
Supplement: Supplementary file 4 — reporting summary [file 43856_2025_830_MOESM4_ESM.pdf]

nature portfolio

Corresponding author(s): DBPR - COMMSMED-24-0705

Last updated by author(s): Nov 21, 2024

Reporting Summary

Nature Portfolio wishes to improve the reproducibility of the work that we publish. This form provides structure for consistency and transparency in reporting. For further information on Nature Portfolio policies, see our Editorial Policies and the Editorial Policy Checklist.

Statistics

For all statistical analyses, confirm that the following items are present in the figure legend, table legend, main text, or Methods section.

n/a	Confirmed	
☐	☒	The exact sample size (*n*) for each experimental group/condition, given as a discrete number and unit of measurement
☐	☒	A statement on whether measurements were taken from distinct samples or whether the same sample was measured repeatedly
☐	☒	The statistical test(s) used AND whether they are one- or two-sided<br>*Only common tests should be described solely by name; describe more complex techniques in the Methods section.*
☒	☐	A description of all covariates tested
☐	☒	A description of any assumptions or corrections, such as tests of normality and adjustment for multiple comparisons
☐	☒	A full description of the statistical parameters including central tendency (e.g. means) or other basic estimates (e.g. regression coefficient) AND variation (e.g. standard deviation) or associated estimates of uncertainty (e.g. confidence intervals)
☐	☒	For null hypothesis testing, the test statistic (e.g. *F*, *t*, *r*) with confidence intervals, effect sizes, degrees of freedom and *P* value noted<br>*Give P values as exact values whenever suitable.*
☒	☐	For Bayesian analysis, information on the choice of priors and Markov chain Monte Carlo settings
☒	☐	For hierarchical and complex designs, identification of the appropriate level for tests and full reporting of outcomes
☒	☐	Estimates of effect sizes (e.g. Cohen's *d*, Pearson's *r*), indicating how they were calculated

Our web collection on statistics for biologists contains articles on many of the points above.

Software and code

Policy information about availability of computer code

Data collection	Flow cytometry data was collected using FacsDiva v8
Data analysis	All statistical tests were performed with GraphPad Prism v10. Glycopeptide data was processed with GlycoPIQ v2.1 and MatchRx software. Flow cytometry data was and analyzed using FlowJo v10.

For manuscripts utilizing custom algorithms or software that are central to the research but not yet described in published literature, software must be made available to editors and reviewers. We strongly encourage code deposition in a community repository (e.g. GitHub). See the Nature Portfolio guidelines for submitting code & software for further information.

Data

Policy information about availability of data

All manuscripts must include a data availability statement. This statement should provide the following information, where applicable:

- Accession codes, unique identifiers, or web links for publicly available datasets
- A description of any restrictions on data availability
- For clinical datasets or third party data, please ensure that the statement adheres to our policy

The original contributions presented in the study are included in the article/supplementary materials, further inquiries can be directed to the corresponding author.

Human research participants

Policy information about studies involving human research participants and Sex and Gender in Research.

Reporting on sex and gender	*Use the terms sex (biological attribute) and gender (shaped by social and cultural circumstances) carefully in order to avoid confusing both terms. Indicate if findings apply to only one sex or gender; describe whether sex and gender were considered in study design whether sex and/or gender was determined based on self-reporting or assigned and methods used. Provide in the source data disaggregated sex and gender data where this information has been collected, and consent has been obtained for sharing of individual-level data; provide overall numbers in this Reporting Summary. Please state if this information has not been collected. Report sex- and gender-based analyses where performed, justify reasons for lack of sex- and gender-based analysis.*
Population characteristics	*Describe the covariate-relevant population characteristics of the human research participants (e.g. age, genotypic information, past and current diagnosis and treatment categories). If you filled out the behavioural & social sciences study design questions and have nothing to add here, write "See above."*
Recruitment	*Describe how participants were recruited. Outline any potential self-selection bias or other biases that may be present and how these are likely to impact results.*
Ethics oversight	*Identify the organization(s) that approved the study protocol.*

Note that full information on the approval of the study protocol must also be provided in the manuscript.

Field-specific reporting

Please select the one below that is the best fit for your research. If you are not sure, read the appropriate sections before making your selection.

☒ Life sciences ☐ Behavioural & social sciences ☐ Ecological, evolutionary & environmental sciences

For a reference copy of the document with all sections, see nature.com/documents/nr-reporting-summary-flat.pdf

Life sciences study design

All studies must disclose on these points even when the disclosure is negative.

Sample size	Ten C57BL/6 female mice of 6-8 weeks old, per group (Total= 70) were used for the present study.
Data exclusions	N/A
Replication	Seven groups of mice (n=10 per group) were treated identically, being immunized with the indicated vaccine formulations (or left as naive) to evaluate the immunogenicity of the antigens indicated.
Randomization	Mice were randomly assigned to various groups upon arrival. No factors were taken into consideration when deciding which group to assign them to.
Blinding	Investigators were blinded and not aware of nature of treatment per group when collecting/analyzing data.

Reporting for specific materials, systems and methods

We require information from authors about some types of materials, experimental systems and methods used in many studies. Here, indicate whether each material, system or method listed is relevant to your study. If you are not sure if a list item applies to your research, read the appropriate section before selecting a response.

Materials & experimental systems

n/a	Involved in the study	
☐	☒	Antibodies
☐	☒	Eukaryotic cell lines
☒	☐	Palaeontology and archaeology
☐	☒	Animals and other organisms
☒	☐	Clinical data
☒	☐	Dual use research of concern

Methods

n/a	Involved in the study	
☒	☐	ChIP-seq
☐	☒	Flow cytometry
☒	☐	MRI-based neuroimaging

Antibodies

Antibodies used

For ELISA, horseradish peroxidase-conjugated secondary antibody was goat anti-mouse (Sigma A2304). A panel of 'homemade' single domain or variable domain of the heavy-chain-only (VHH) antibodies were developed and described in a previously published study. They are used in Figure 4B and a sdAb (NRCoV-2 11) is used for the SPR in Figure 4A. See here for the original study: Rossotti, M.A., van Faassen, H., Tran, A.T. et al. Arsenal of nanobodies shows broad-spectrum neutralization against SARS-CoV-2 variants of concern in vitro and in vivo in hamster models. Commun Biol 5, 933 (2022). https://doi.org/10.1038/s42003-022-03866-z

Validation

See here for the manuscript validating these nanobodies: Rossotti, M.A., van Faassen, H., Tran, A.T. et al. Arsenal of nanobodies shows broad-spectrum neutralization against SARS-CoV-2 variants of concern in vitro and in vivo in hamster models. Commun Biol 5, 933 (2022). https://doi.org/10.1038/s42003-022-03866-z

Eukaryotic cell lines

Policy information about cell lines and Sex and Gender in Research

Cell line source(s)

CHO-2353 is described by our group in the following manuscript : Poulain, A., et al., Rapid protein production from stable CHO cell pools using plasmid vector and the cumate gene-switch. J Biotechnol, 2017. 255: p. 16-27.
HEK293-ACE2 cells BEI Resources repository of ATCC and the NIH (NR-52511)
HEK293-hACE2/TMPRSS2 cells BEI Rescources repository of ATCC and the NIH (NR-55293)

Authentication

HEK293 ACE2 and HEK293-hACE2/TMPRSS2 cell morphology was authenticated via microscopy.

Mycoplasma contamination

We confirmed all cells used in this study are Mycoplasma free.

Commonly misidentified lines
(See ICLAC register)

Name any commonly misidentified cell lines used in the study and provide a rationale for their use.

Animals and other research organisms

Policy information about studies involving animals; ARRIVE guidelines recommended for reporting animal research, and Sex and Gender in Research

Laboratory animals

Female C57BL/6 mice (6-8 weeks old) were purchased from Charles River Laboratories (Saint-Constant, Canada).

Wild animals

N/A

Reporting on sex

Findings do not apply to a particular sex. Immune responses to these type of vaccine formulations have been previously shown to be similar in male and female murine models.

Field-collected samples

N/A

Ethics oversight

Animals were maintained at the animal facility of the National Research Council Canada (NRC) in accordance with the guidelines of the Canadian Council on Animal Care. All procedures performed on animals in this study were approved by our Institutional Review Board (NRC Human Health Therapeutics Animal Care Committee) and covered under animal use protocol 2020.10. All experiments were carried out in accordance with the ARRIVE guidelines.

Note that full information on the approval of the study protocol must also be provided in the manuscript.

Flow Cytometry

Plots

Confirm that:

- [ ] The axis labels state the marker and fluorochrome used (e.g. CD4-FITC).
- [ ] The axis scales are clearly visible. Include numbers along axes only for bottom left plot of group (a 'group' is an analysis of identical markers).
- [ ] All plots are contour plots with outliers or pseudocolor plots.
- [ ] A numerical value for number of cells or percentage (with statistics) is provided.

Methodology

Sample preparation

Indicated dilutions of mouse serum were mixed with 250ng of biotinylated spike and 1×10^5 HEK-293T-hACE2 cells and incubated for 1 hour in the fridge protected from light. Samples were washed with PBS + 1% BSA + 0.05% azide twice prior to incubating with streptavidin-phycoerythrin for 1 hour in the fridge protected from light. Samples were again washed twice with PBS + 1% BSA + 0.05% azide prior to fixation. Once fixed, samples were stored in the fridge until analyzed on the flow cytometer.

Instrument	LSR Fortessa SORP
Software	FacsDiva v8 for software acquisition, FlowJo v10 for data analysis
Cell population abundance	100%; all HEK293T ACE2 cells bound spike and were all important
Gating strategy	Cell population was first identified via FSC/SSC gating and singlets were identified via FSC-A / FSC-H gating on the linear dimension to remove doublets. The singlet population was considered for all geometric mean fluorescent intensity parameters of phycoerythrin that were output for analysis.

☐ Tick this box to confirm that a figure exemplifying the gating strategy is provided in the Supplementary Information.
